# Impacts of Sandwich Caregiving on Labor Market Outcomes

**DOI:** 10.1093/geroni/igaf122.1436

**Published:** 2025-12-31

**Authors:** Siavash Radpour, Aida Farmand, Jessica Forden

**Affiliations:** Stockton University, Galloway, New Jersey, United States; Office of the New York City Comptroller, New York, New York, United States; The New School, New York, New York, United States

## Abstract

This study examines the dynamics of caregiving and its impact on labor market outcomes, with a focus on the often-overlooked segment of sandwich caregivers defined as individuals engaged in both childcare and adult care. Utilizing data from the American Time Use Survey (ATUS) spanning the years 2012 to 2022, our findings reveal significant trends: women of all ages are more likely than men to be sandwich caregivers, individuals with higher educational attainment tend to assume sandwich caregiving roles later in life, and black workers are more inclined to provide sandwich care at an earlier age. Inverse Probability Weighted Regression Adjustment (IPWRA) is used to estimate unbiased treatment effects while propensity score matching is utilized to model interactions between sandwich caregiving and gender. Results indicate that sandwich caregivers are 5.7 percent less likely to participate in the labor force and, for those employed, work 5 hours less per week compared to non-caregiving workers, with more pronounced effects on women. This research contributes to a deeper understanding of the impacts of sandwich caregiving responsibilities on extensive and intensive margins, highlighting the need for policies that address the unique challenges faced by sandwich caregivers.

